# Floral Scent Emission from Nectaries in the Adaxial Side of the Innermost and Middle Petals in *Chimonanthus praecox*

**DOI:** 10.3390/ijms19103278

**Published:** 2018-10-22

**Authors:** Zhineng Li, Yingjie Jiang, Daofeng Liu, Jing Ma, Jing Li, Mingyang Li, Shunzhao Sui

**Affiliations:** 1 College of Horticulture and Landscape Achitecture, Southwest University, Chongqing 400715, China; znli@swu.edul.cn (Z.L.); sherneole@163.com (Y.J.); liu19830222@163.com (D.L.); majing427@swu.edu.cn (J.M.); acejing@126.com (J.L.); limy@swu.edu.cn (M.L.); 2 Key Laboratory of Horticulture Science for Southern Mountains Regions, Ministry of Education, Chongqing 400715, China; 3 Chongqing Engineering Research Center for Floriculture, Chongqing 400715, China

**Keywords:** *Chimonanthus praecox*, nectary, floral scent, gene expression

## Abstract

Wintersweet (*Chimonanthus praecox*) is a well-known traditional fragrant plant and a winter-flowering deciduous shrub that originated in China. The five different developmental stages of wintersweet, namely, flower-bud period (FB), displayed petal stage (DP), open flower stage (OF), later blooming period (LB), and wilting period (WP) were studied using a scanning electron microscope (SEM) to determine the distribution characteristics of aroma-emitting nectaries. Results showed that the floral scent was probably emitted from nectaries distributed on the adaxial side of the innermost and middle petals, but almost none on the abaxial side. The nectaries in different developmental periods on the petals differ in numbers, sizes, and characteristics. Although the distribution of nectaries on different rounds of petals showed a diverse pattern at the same developmental periods, that of the nectaries on the same round of petals showed some of regularity. The nectary is concentrated on the adaxial side of the petals, especially in the region near the axis of the lower part of the petals. Based on transcriptional sequence and phylogenetic analysis, we report one nectary development related gene *CpCRC* (*CRABS CLAW*), and the other four YABBY family genes, *CpFIL* (*FILAMENTOUS FLOWER*), *CpYABBY2*, *CpYABBY5-1*, and *CpYABBY5-2* in *C*. *praecox* (accession no. MH718960-MH718964). Quantitative RT-PCR (qRT-PCR) results showed that the expression characteristics of these YABBY family genes were similar to those of 11 floral scent genes, namely, *CpSAMT*, *CpDMAPP*, *CpIPP*, *CpGPPS1*, *CpGPPS2*, *CpGPP*, *CpLIS*, *CpMYR1*, *CpFPPS*, *CpTER3*, and *CpTER5*. The expression levels of these genes were generally higher in the lower part of the petals than in the upper halves in different rounds of petals, the highest being in the innermost petals, but the lowest in the outer petals. Relative expression level of *CpFIL*, *CpCRC*, *CpYABBY5-1*, and *CpLIS* in the innermost and middle petals in OF stages is significant higher than that of in outer petals, respectively. SEM and qRT-PCR results in *C. praecox* showed that floral scent emission is related to the distribution of nectaries.

## 1. Introduction

Nectaries are glandular structures that secrete nectar, a carbohydrate-rich solution that is composed mainly of sugars and it generally serves as a reward for pollinators or for as protectors (e.g., ants) against herbivores, or, as a lure for animal prey in carnivorous plants [1]. Nectaries are most wide spread in angiosperms, particularly within flowers, and in ferns and Gnetales [2]. Arabidopsis *CRC* is expressed in the nectary throughout its development and plays a role in the specification and/or differentiation of the nectary [3]. *CRC* is also responsible for carpel growth and fusion, and floral meristem termination [2,4,5]. *CRC* encodes a putative transcription factor of the YABBY gene family, which also includes FIL, INO (INNER NO OUTER), YAB2 (YABBY2), YAB3, and YAB5 [6,7]. The YABBY family is characterized in *Arabidopsis* and rice [2,4,6,8,9,10,11,12,13,14]. INO expression occurs only in the abaxial domain of the ovule integument [4]. The “vegetative YABBYs” (FIL, YAB2, and YAB5) in angiosperm are exclusively expressed in leaf-homologous organs, both vegetative and floral, they are involved in leaf development, such as the leaf margin establishment that guides laminar growth and leaflet initiation; maintenance of leaf polarity; and, activation of leaf maturation processes and repression of shoot apical meristem genes [7]. Petunia *PhCRC1*/*2* expressed in developing nectaries and carpels, similar to *Arabidopsis CRC* expression [15,16]. No nectary glands develop in *crc* mutants [17]. Locations of nectaries are highly variable in broader taxonomic terms [18], although their locations within flowers are constant at the family level. Nectaries tend to be associated with the perianth in basal angiosperms [19], while they are usually associated with carpels and stamens in the eudicots. Fahn argued that nectaries position within flowers trends to shift from peripheral perianth positions in basal taxa to central positions that are associated with reproductive organs in more derived taxa [20].

Wintersweet (*C. praecox*) is a unique traditional deciduous woody flower that is popularly used in floral arrangement, bonsai growing, and landscaping in many countries because of its unique flowering time and distinctive fragrance in deep winter. Wintersweet is a potential spice material because of its volatile aromatic substances and can be used in perfumery, cosmetics, aromatic tea, aromatherapy, and the food industry [21,22,23].

More than 30 floral scent volatiles have been detected in *C*. *praecox* flowers, consisting almost exclusively of volatile benzenoids and terpenoids (monoterpenes and sesquiterpenes) [24,25]. Terpenoids play a leading role among these volatiles. These compounds in wintersweet have minimal molecular-genetic characterization, except for the homologous genes of *CpFPPS* and *CpSAMT* [22,26].

Here, we reported the distribution characteristics of nectaries in *C. praecox* and the expression profiles of nectary development related gene *CpCRC,* the other four *YABBY* family genes (*CpFIL*, *CpYABBY2*, *CpYABBY5-1*, and *CpYABBY5-2*) and floral scent related genes to help understand floral scent origination and the molecular regulation of nectary development in wintersweet and other plants of scented flowers.

## 2. Results

### 2.1. Distribution Characteristics of Nectary on Petals of Different Stages

**Figure 1:**
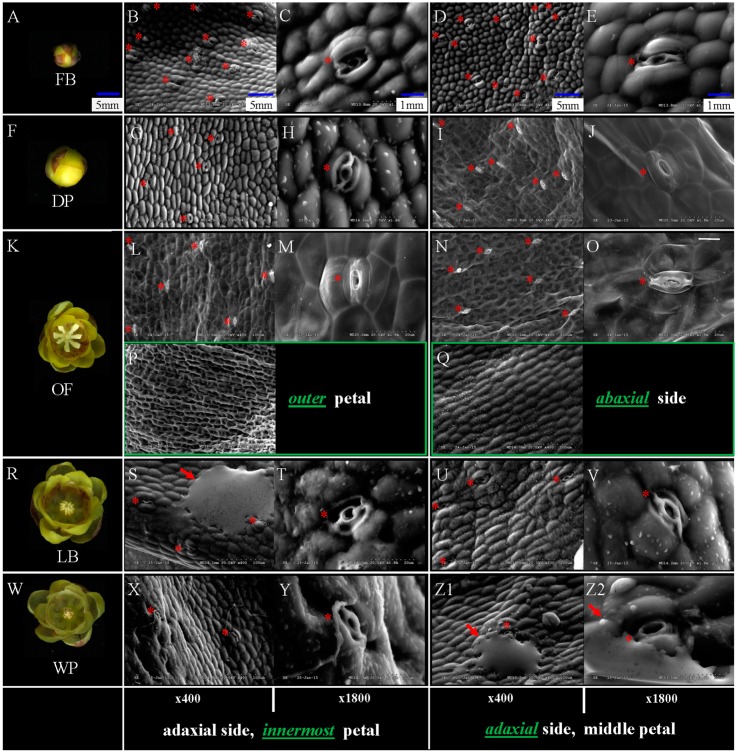
Morphology of the surface of petal glands on different stages in the *Chimonanthus praecox* by SEM. (**A**,**F**,**K**,**R**,**W**)floral meristem of five different developmental stages (FB, DP, OF, LB, and WP); (**B**,**C**;**G**,**H**;**L**,**M**;**S**,**T**;**X**,**Y**) nectaries on adaxial side of innermost petals in five different developmental stages. (**D**,**E**;**I**,**J**;**N**,**O**;**U**,**V**;**Z1**,**Z2**) nectaries on the adaxial side of middle petals in five different developmental stages. (**B**,**D**,**G**,**I**,**L**,**N**,**S**,**U**,**X**,**Z1**) surface of the glandular tissue, showing the nectary stomata (red asterisk). (**C**,**E**,**H**,**J**,**M**,**O**,**T**,**V**,**Y**,**Z2**) close-up of the nectary stomata. (**P**,**Q**) (in green box) no nectary distribution on adaxial side of outer petal and abaxial side of middle petals in OF stages. Scale bar is the same in (**A**,**F**,**K**,**R**,**W**); (**B**,**D**,**G**,**I**,**L**,**N**,**P**,**Q**,**S**,**U**,**X**,**Z1**); and (**C**,**E**,**H**,**J**,**M**,**O**,**T**,**V**,**Y**,**Z2**), respectively. Red arrow shows the substance of floral scent. The magnification was ×400 (left) and ×1800 (right), respectively.

SEM analysis was performed to determine the distribution characteristics of nectary on petals in different stages of petals in different developmental stages of floral meristem (FB, DP, OF, LB, and WP), and receptacle, pistil, and stamen (Figure 1A,F,K,R,W). The nectaries were mainly located in the adaxial side of the innermost and middle petals (red asterisk shown, Figure 1B–E,G–J,L–O,S–V,X–Z2). The adaxial/abaxial side of the outer petals and the abaxial side of innermost/middle petals of different stages had no nectary distribution. Figure 1P,Q show the adaxial and abaxial sides of the outer and middle petals in the OF stages (in green box), respectively, which there is no nectary detected at all. The numbers of nectaries changed in the FB to WP during flower senescence. The numbers of nectaries were equal or slightly higher in the middle petal than that in the innermost petal under the same magnification (×400, Figure 1). Morphological difference in different stages of nectaries are shown in Figure 1. With the development of floral meristem, the length-width ratio of nectaries became small and evaginated (×1800, Figure 1). The substance of floral scent was found beside the nectaries (red arrow shown, Figure 1S,Z1,Z2). The cell size increased from FB and DP to OF with the development of floral meristem. This phenomenon is similar to that in LB and WP. Larger number of nectaries was observed in FB or DP than in OF at the same magnification (×400) because of the different cell sizes in different development stages (Figure 1B,D,G,I,L,N). The number of nectaries in OF was higher than that in LB or WP (Figure 1L,N,S,U,X,Z1), with similar cell size in three different development stages.

**Figure 2:**
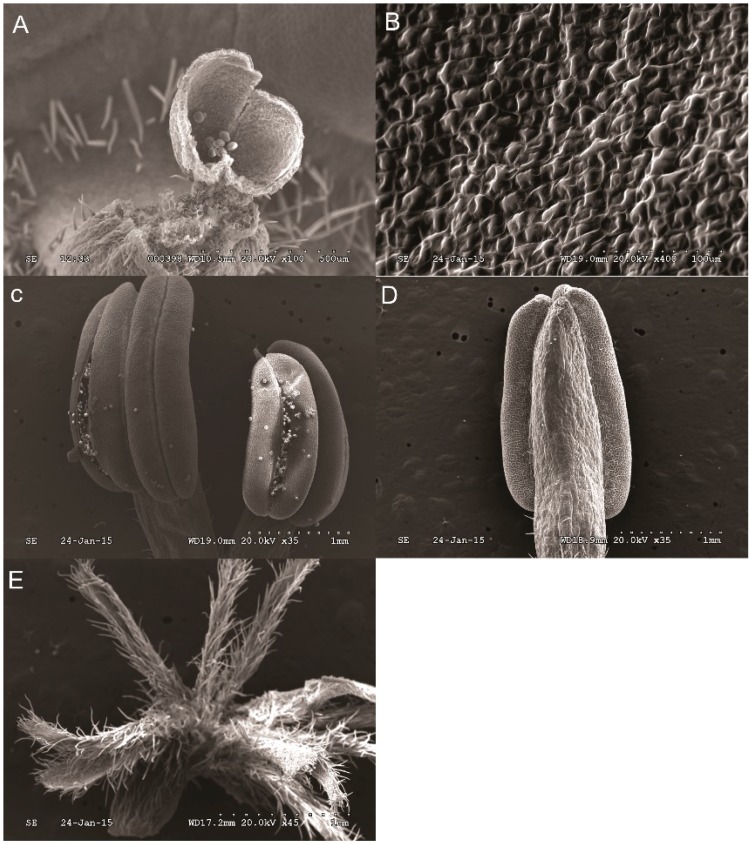
No nectaries distribution of the stamen and pistil in OF in the *Chimonanthus praecox* by SEM. (**A**), receptacle; (**B**), perianth; (**C**), front of stamen; (**D**), back of stamen; and, (**E**), pistil.

**Figure 3:**
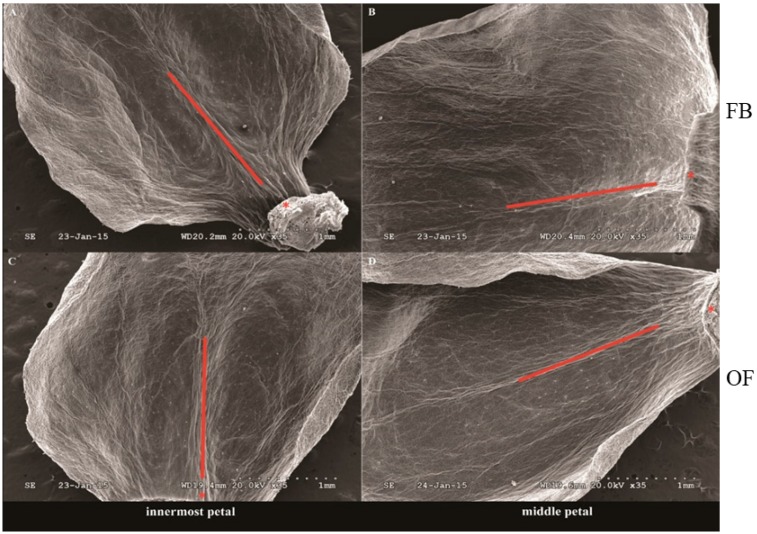
Distribution of the nectaries in FB and OF in the *Chimonanthus praecox* by SEM. Little white dot shown the nectaries concentrated near the axis of petals. Red line represented the axis of the petal. Red asterisks show the bottom of the petal.

No nectary was detected on the receptacle, perianth, stamen, and pistilin all five stages (FB, DP, OF, LB, and WP) (just show results in OF stage, Figure 2). Concentrated nectaries were found in the region near the axis of the lower part of petals, but almost none in the upper and edge of petals (Figure 3 just show the stages of FB and OF). Nectaries are not uniformly distributed in the petals (Figure 1B,D,G,I,L,N,S,U,X,Z1 and Figure 3). These distribution characteristics of nectaries were perhaps related to the *YABBY* gene family, which controls the build of nectaries development and dorsiventral polarity.

### 2.2. Sequence Alignment and Phylogenetic Analysis

**Figure 4:**
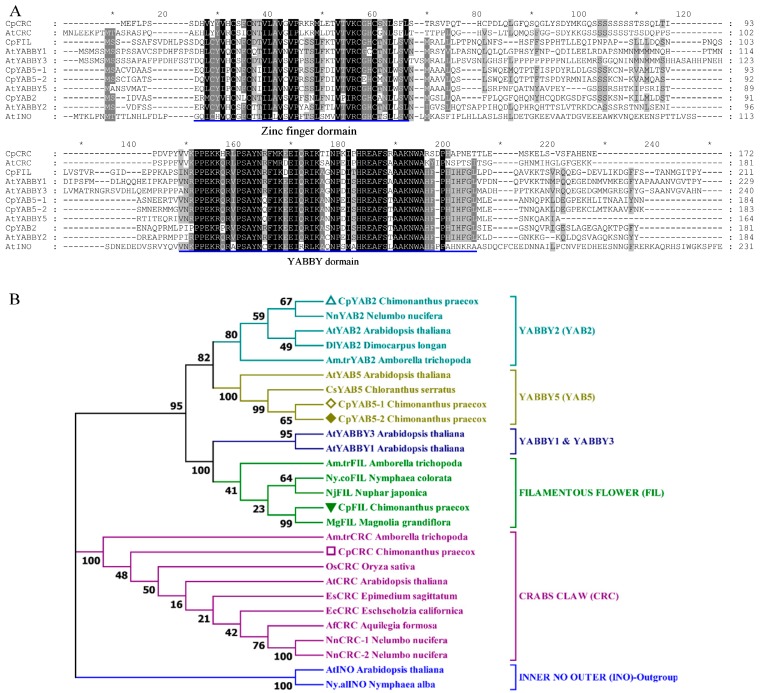
Sequence alignment and phylogenetic analysis of YABBY proteins. (**A**) Sequence alignment of YABBY proteins in *C*. *praecox* and *A*. *thaliana*. Conserved domains (Zinc finger domain and YABBY domain) are underlined in blue. Identical residues are highlighted in black and similar residues are highlighted in grey. Dotted line and asterisk represented the gap and the position of odd times of ten in protein sequence. (**B**) Phylogenetic analysis by Neighbor-joining (NJ) bootstrap analysis (1000 replications). AtINO (*A*. *thaliana*) and Ny.alINO (*Nymphaea alba*) as outgroup. The gene accession number is shown in Materials and Methods. Five different symbols in front of the protein name reprent the five YABBY protein of *Chimonanthus praecox* in this study.

Partial or complete *CpFIL*, *CpCRC*, *CpYABBY2*, *CpYABBY5-1*, and *CpYABBY5-2* cDNAs contain open reading frame of 636, 519, 546, 555, and 552bp, respectively. The predicted CpFIL, CpCRC, CpYABBY2, CpYABBY5-1, and CpYABBY5-2 proteins of 212, 173, 182, 185, and 184 amino acid residues contains a zinc-finger domain in the N-terminus and a YABBY domain in the C-terminus (Figure 3A). The putative CpFIL protein shares 89% and 82% similarity with MgFIL from *Magnolia grandiflora* and Ny.coFIL from *Nymphaea colorata*. CpCRC protein shows 67% and 59% identity with the products of NnCRC-1/2 in *Nelumbo nucifera* and AtCRC in *Arabidopsis*. CpYABBY2 protein shares 72% and 57% similarity with NnYAB2 from *N. nucifera* and AtYAB2 from *Arabidopsis*, respectively. The putative CpYABBY5-1 protein shared high similarity (81%) with CpYABBY5-2, and they both showed 89% and 93% identity with the products of CsYAB5 in *Chloranthus serratus*, and 72% and 69% with that of AtYABBY5 in *Arabidopsis* (Figure 4A and Figure S1; Table S1).

The sequences of the five YABBYs protein homologues were aligned with the respective FIL, CRC, YABBY2, YABBY5-1, and YABBY5-2 proteins of the multiple angiosperm taxa for phylogenetic analysis. The CpYAB2 homologue clustered with non-core NnYAB2 of *Nelumbo nucifera*, DlYAB2 of *Dimocarpus longan*, Am.trYAB2 from *Amborella trichopoda* and core AtYAB2 from *A*. *thaliana*. The two YABBY5 sequences from *C. praecox*, CpYAB5-1 and CpYAB5-2, formed a sister group to the basal eudicot, CsYAB5 of *Chloranthus serratus*, formed a clade YABBY5 with AtYAB5 of *A. thaliana*. The CpFIL sequence aligned near to none-core basal eudicots, MgFIL of *Magnolia grandiflora,* NjFIL of *Nuphar japonica*, *Nymphaea colorata* Ny.coFIL and *A. trichopoda* Am.trFIL, and formed FIL clade. Together with *A*. *thaliana* AtCRC, the CpCRC, *A. trichopoda* Am.trCRC, *Oryza sativa* OsCRC, EsCRC of *Epimedium sagittatum*, EcCRC of *Eschscholzia californica*, *N. nucifera* NnCRC-1, and NnCRC-2 formed a clade of CRC (Figure 4B).

### 2.3. Expression Analysis

**Figure 5:**
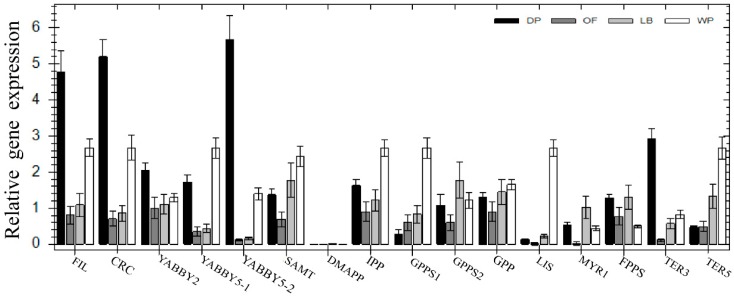
Quantitative real-time PCR analysis of different genes in four different developmental stages of DP, OF, LB, and WP. *Tublin* homologous gene of *Chimonanthus praecox* was used as an internal control.

In order to illustrate the correlation between nectary development and gene expression of *CpCRC* and the other four YABBY genes, also that of the nectary development and the floral scent, heatmap analysis with the RNA-Seq database in DP, OF, and WP stages [27] and qRT-PCR were conducted using cDNA derived from DP, OF, LB, and WP to determine the expression profile of five YABBY genes and 11 floral scent related genes in the flower buds of different developmental stages in *C*. *praecox* (Figure 1F,K,R,W). Almost no *CpDMAPP* was detected in DP, OF, LB, and WP. The relative expression of one nectary development related gene *CpCRC* and the other four YABBY family genes (*CpFIL*, *CpYABBY2*, *CpYABBY5-1*, and *CpYABBY5-2*) and seven floral scent genes (*CpSAMT*, *CpIPP*, *CpGPPS1*, *CpGPP*, *CpLIS*, *CpTER3*, and *CpTER5*) gradually increased in OF to LB and WP. The expression levels of *CpFIL*, *CpCRC*, *CpYABBY2*, *CpYABBY5-2*, and *CpTER3* were the highest, and those of *CpGPPS1* and *CpTER5* were the lowest in DP. The expression of *CpYABBY5-1*, *CpSAMT*, *CpIPP*, *CpGPP*, and *CpLIS* in DP was higher than that in OF but lower than that in WP. The highest and lowest expression levels of *CpGPPS2* and *CpMYR1* were in LB and OF, respectively. The expression of *CpFPPS* in DP was similar to that in LB, higher than that in OF, and lowest in WP (Figure 5).

**Figure 6:**
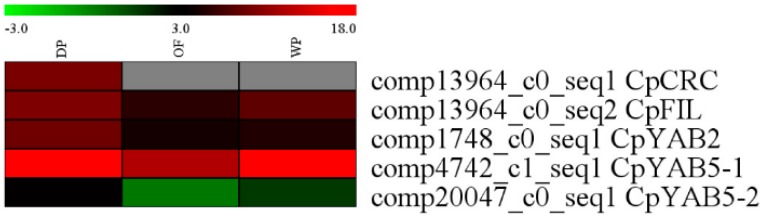
Heatmap analysis of 5 YABBY family gene expression in DP, OF, and WP stages.

According to the RNA-Seq database in DP, OF and WP stages, *CpFIL*, *CpCRC*, *CpYABBY2*, *CpYABBY5-1*, and *CpYABBY5-2* have similar expression pattern. The expression level in DP was higher than that in OF and WP and the lowest in WP (Figure 6).

**Figure 7:**
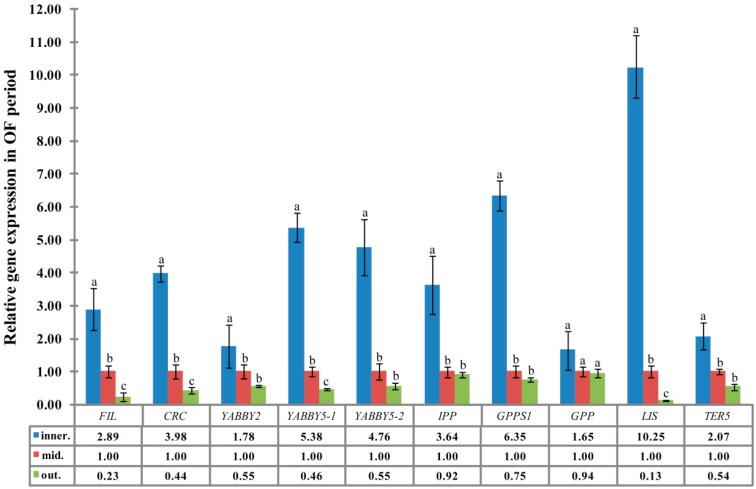
Quantitative real-time PCR analysis of different genes in three different rounds of petals in OF stages. Inner., mid., and out. represent innermost, middle, and outer petals, respectively. *Tublin* homologous gene of *C*. *praecox* was used as aninternal control. (Notes: *t*-test used for significant difference analysis; data is the means of relative expression; a, b, c show *p* < 0.05 significant level).

To further clarify the correlation between the distribution characteristics of nectaries in three different round of petals and gene expression, the expression profile of five YABBY genes and five floral scent related genes in the innermost, middle, and outer petals in OF stages (Figure 1K) was detected using qRT-PCR. The expression level of 5 YABBY genes and five floral scent-related genes gradually decreased in the innermost to the middle and outer petals. The relative expression levels of *CpFIL*, *CpCRC*, *CpYABBY2*, *CpYABBY5-1*, and *CpYABBY5-2* in the innermost petals were 1.78- to 5.38-fold, and 3.24- to 12.57-fold higher than that in the middle and the outer petals, respectively. The relative expression levels of floral scent genes (*CpIPP*, *CpGPPS1*, *CpGPP*, *CpLIS*, and *CpTER5*) in the innermost petals were approximately 1.65- to 10.25-fold higher than those in the middle petals and 1.76- to 78.85-fold higher than those in the outer petals (Figure 7). They all have significant difference between the relative expression level of *CpFIL*, *CpCRC*, *CpYABBY5-1*, and *CpLIS* in middle petals and that of in outer petals (Figure 7).

**Figure 8:**
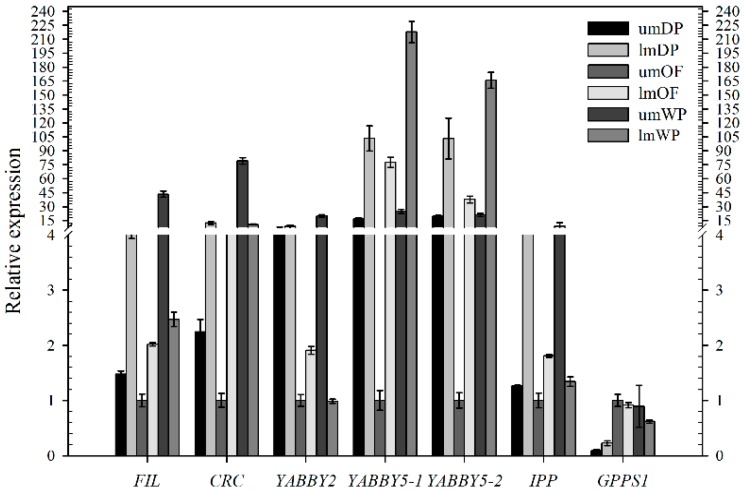
Quantitative real-time PCR analysis of different genes in the upper and lower halves of middle petals in DP, OF, and WP stages. *Tublin* homologous gene of *C*. *praecox* was used as an internal control. “um” and “lm” represent upper and lower half of middle petals, respectively.

Based on SEM results, for the sake of the relationship between the distridution characteristics of nectaries in the same round of petals and the gene expression, qRT-PCR was conducted using cDNA derived from the upper and lower halves in middle petals from DP, OF, and WP (Figure 1F,K,W) to further detect the expression profile of one nectary development related gene *CpCRC*, four YABBY family genes (*CpFIL*, *CpYABBY2, CpYABBY5-1*/*2*) and two floral scent genes (*CpIPP* and *CpGPPS1*). The relative expression of these five YABBY family genes and *CpIPP* had a similar expression pattern. The expression levels in the lower half of middle petals were higher than those in the upper halves in DP and OF stages, including the *CpYABBY5-1* and *CpYABBY5-2* in the WP stage and *CpGPPS1* in the DP stage. However, the relative expression levels of *CpFIL*, *CpCRC*, *CpYABBY2*, *CpIPP,* and *CpGPPS1* in the lower half of middle petals were lower than those in the upper halves in WP stage and were similar to those of *CpGPPS1* in the OF stage (Figure 8).

## 3. Discussion

Results of SEM analysis in *C*. *praecox* show that nectaries were distributed on the adaxial side of the innermost and middle petals but not on the abaxial side. No nectary was detected in all five stages (FB, DP, OF, LB, and WP) on the outer petals, including in the receptacle, perianth, stamen, and pistil. The surface morphology of the innermost and middle glands of *C*. *praecox* is similar to that of the inner petal glands of *Alphonsea glandulosa* and *Petunia* [16,28]. The surface of the nectar glands is different from the surrounding epidermis, and nectar stomata are found across the surface of the glandular tissues [28]. The nectar stomata are raised slightly above the epidermis with an aperture for nectar secretion [29]. Although the locations of nectaries within flowers vary highly in terms of broader taxonomic terms, their locations are constant at the family level [30]. Nectaries are usually associated with carpels and stamens in eudicots, but are related to perianth in basal angiosperms [19]. *C. praecox* belongs to Calycanthaceae, Laurales, Magnoliids, and is clustered to Magnoliales, Piperales, and Canellales, which are close to Chloranthales, Austrobaileyales, Nymphaeales, and Amborellales [31]. In Magnoliidae, *C. praecox* has no nectary distribution on its receptacle, perianth, stamen, or pistil, but has some on the adaxial side of the innermost and middle petals; this finding partly supports that of a previous study, nectaries position within flowers trends to shift from peripheral perianth in basal taxa to central reproductive organs in more derived taxa [19,20].

Monoterpenes, such as myrcene, geraniol, linalool and sesquiterpene compounds, are the main aroma components of *C. praecox* [32,33]. Therefore, the concentration of universal precursor of monoterpene (GPP) and its substrate IPP can indirectly reflect the aroma production of *C. praecox*. qPCR analysis of *CpIPP* and *CpGPPS* gene in different parts of petals can indirectly determine the location of aroma substances. The expression levels of the nectary development related genes *CpCRC*, the other four YABBY family genes (*CpFIL*, *CpYABBY2,* and *CpYABBY5-1*/*2*) and five floral scent genes (*CpIPP*, *CpGPPS1*, *CpGPP*, *CpLIS*, and *CpTER5*) in the innermost petals of *C. praecox* were higher than those in the middle and outer petals, but they were the lowest in the outer petals. The *CpLIS* expression was increased seven-fold at the OF stage, which is responsible for α-linalool biosynthesis [27]; and α-linalool accounts for 36% of the total quantity of volatile compounds has been reported in wintersweet flowers [34]. The expression results were consistent with the characteristics of nectary distribution based on SEM analysis (Figure 1 and Figure 7). 

The expression pattern of five YABBY genes in *C*. *praecox* by qPCR was in accordance with the RNA-Seq in DP, OF, and WP stages (Figure 5 and Figure 6). The expression levels of *CpIPP* and *CpGPPS* were significantly different in the different halves of the petals; those in the upper halves were significantly lower than those in the lower halves during the first two periods. This result is consistent with that of SEM analysis (Figure 1), which stated that the nectaries were mainly distributed in the lower half part of the petals near the axis and were rarely distributed on the edge and upper half part of the petals. Nectaries are not uniformly distributed in the petals, that is why the numbers of nectaries were equal or slightly higher in the middle petal than that in the innermost (×400, Figure 1). The expression characteristics of *CpFIL*, *CpCRC*, *CpYABBY2*, and *CpYABBY5-1*/*2* were generally similar to those of *CpIPP* and *CpGPPS*.

At least one YABBY gene family member *CpCRC* was expressed in all asymmetric above-ground organs in a polarity, suggesting that this gene is involved in establishing dorsiventral polarity in all of these organs. The YABBY gene family controls the build of dorsiventral (abaxial/adaxial) polarity [4,10,35]. Therefore, we proposed that the floral scent mainly originates from the nectaries that are distributed neither on the abaxial side of the innermost and middle petals nor on the outer petals, but on the adaxial side of the innermost and middle petals. This unbalanced distribution of the nectaries is caused by dorsiventrality differentiation, one of the most important polarities in the development of lateral organs in plants.

## 4. Materials and Methods

### 4.1. Plant Material

*C. praecox* plants of 21-years old were grown in the campus of Southwestern University (106°43′ E, 29°83′ N, Beibei District, Chongqing City, China) under natural photoperiod. Flower development was divided into the following five stages: FB is the stage wherein the flower bud is closed, and the petals are yellow; DP wherein the petals unroll; OF wherein the petals reach full opening, and the stamens bent toward the adaxial side of innermost petals and away from the pistils at a right angle; LB that occurs after two days of OF, where the stamens commence to move to enclose the pistils; and WP wherein the flower is pollinated, and the petals and stamens start to wilt. Floral tissue samples, such as receptacle, sepals, petals, stamens, and pistils were obtained from five different stages. Some of the petals were divided into upper and lower halves. All plant materials were harvested then fixed with FAA buffer or frozen in liquid nitrogen and stored at −80 °C for RNA extraction.

### 4.2. Scanning Electron Microscope (SEM)

Fresh petals of *C. praecox* were soaked for an hour in pre-cold 2% glutaraldehyde solution and then were rinsed three to four times with 0.1 M phosphate buffer (pH 7.2) for 1 h. The buffer was discarded, and ethanol dehydration was conducted in a step-by-step gradient. Ethanol concentrations were 30%, 50%, 70%, 80%, 90%, and 100% for 25 min each. The alcohol was washed, and 1:1 mixture of isoamyl acetate to ethanol was added. Then, the solution was added with pure isoamyl acetate, soaked for 10–20 min for each step, stirred properly, and air dried before the electron microscope observation.

### 4.3. Sequence Alignment and Phylogenetic Analysis

The sequences of one nectary development related gene *CRC*, four other YABBY family genes (*FIL*, *YABBY2*, *YABBY5-1*/*2*) and 11 floral scent genes in *C. praecox* were selected from the Illumina deep sequencing [27]. Blastn of these genes were obtained and named as *CpFIL*, *CpCRC*, *CpYABBY2*, *CpYABBY5-1*, *CpYABBY5-2*, *CpSAMT*, *CpDMAPP*, *CpIPP*, *CpGPPS1*, *CpGPPS2*, *CpGPP*, *CpLIS*, *CpMYR1*, *CpFPPS*, *CpTER3*, and *CpTER5.* The sequences included in the analysis were downloaded from the NCBI GenBank (http://www.ncbi.nlm.nih.gov). The amino acid sequences of the YABBY family were aligned using ClustalX 1.83 [36]. Neighbor-joining (NJ) bootstrap analysis (1000 replications) with Poisson correction for the amino acids was performed using MEGA 4 [37]. Sequence data for analysis can be found in the GenBank/EMBL databases under the following accession numbers: *CpFIL*, *CpCRC*, *CpYABBY2*, *CpYABBY5-1* and *CpYABBY5-2* from *C. praecox*; *AtINO*, *AtCRC*, *AtYABBY1*, *AtYABBY2*, *AtYABBY3,* and *AtYABBY5* (AAF23754, NP_177078, NP_566037, AF136539, AF136540, NM_179749) from *A. thaliana*; *Am.trCRC*, *Am.trFIL,* and *Am.trYAB2* (AJ877257, AB168113, AB126654) from *Amborella trichopoda*; *AfCRC* (AY854797) from *Aquilegia formosa*; *CsYAB5* (BAF65259) from *Chloranthus serratus*; *DlYAB2* (ACN59438) from *Dimocarpus longan*; *EsCRC* (GH62810) from *Epimedium sagittatum*; *EcCRC* (CAQ17052) from *Eschscholzia californica*; *NnYAB2*, *NnCRC-1*, and *NnCRC-2* (XP_010247861, XM_010259669, XM_010259670) from *Nelumbo nucifera*; *NjFIL* (BAD83708) from *Nuphar japonica*; *Ny.alINO* (AB092980) from *Nymphaea alba*; *Ny.coFIL* (BAF65258) from *Nymphaea colorata*; *MgFIL* (BAF65261) from *Magnolia grandiflora*; and *OsCRC* (AAR84663) from *Oryza sativa*.

### 4.4. Gene Expression Analysis

Tissues sampled for gene expression analysis include flower buds of four different developmental stages (DP, OF, LB, and WP), three different rounds (innermost, middle, and outer) of petals in OF stages, the upper and lower halves of middle petals in DP, OF, and WP stages. Total RNA for the expression analysis was extracted using RNAprep pure kit (Tiangen, Beijing, China) according to the manufacturer’s instructions. Exactly 3 μg of RQ1 RNase-Free DNase (Promega, Madison, WI, USA) pre-treated total RNA was reverse transcribed according to the instructions of the Primescript RT reagent kit (Takara, Tokyo, Japan). qRT-PCR was performed to determine the expression pattern of one nectary development related gene*CpCRC*, four YABBY family genes (*CpFIL*, *CpYABBY2*, *CpYABBY5-1*, and *CpYABBY5-2*) and 11 floral scent genes, such as *CpSAMT*, *CpDMAPP*, *CpIPP*, *CpGPPS1*, *CpGPPS2*, *CpGPP*, *CpLIS*, *CpMYR1*, *CpFPPS*, *CpTER3*, and *CpTER5*.

The primers for qRT-PCR are listed in Table 1. Reactions were performed with the Sso Fast Eva Green Supermix (Bio-Rad, Hercules, CA, USA) and analyzed using Bio-Rad CFX96 (Bio-Rad CFX Manager Software Version 1.6). Thermocycler conditions were 95 °C for 30 s, followed by 40 cycles of 95 °C for 5 s and 60 °C for 5 s. qRT-PCR products were amplified using 5 μL 2× Sso Fast Eva Green Supermix, 0.5 μL RT reaction mixture, 0.5 μL of forward and reverse primer (10 μmol/μL) each, and RNase Free dH_2_O to a final volume of 10 μL. Relative amounts of transcripts were calculated using the comparative CT method (2^−ΔΔ*C*t^), and the values were normalized. The house-keeping gene *CpTublin* of *C. praecox* was used as internal control. Data are shown as mean values ± standard deviation (SD) from three replicates for each sample. Significant difference was carried out by *t*-test (*p* < 0.05).

The expression patterns of the five YABBY genes were estimated by FPKM values and were visualized using MultiExperiment Viewer (Broad Institute of MIT and Harvard University, Boston, MA, USA [38]).

## Figures and Tables

**Table 1:** Primer for real-time PCR.

Gene Name	Forward Primer Sequence	Reverse Primer Sequence
*Actin*	AGGCTAAGATTCAAGACAAGG	TTGGTCGCAGCTGATTGCTG
*CpFIL*	AATCCCGACATAACCCACAGAGAG	TCCTGTTGGCGCACGCTAGTT
*CpCRC*	CCTCCCGTCACCTTACAAACTACAG	CTGCTACAAGGAACACTGACCGC
*CpYABBY2*	CCATTGTCAAGATAAAGGTAGCGATT	CTGGTGGTGGTATAGGTAGCATTCG
*CpYABBY5-1*	TCTCCCTCTCTATTTATCCTCGTTT	GTAAAAGGCTAAAGCAGGATCATG
*CpYABBY5-2*	TTTTGAACACTGGAAACTTCGTCTT	GATGCAGCTCGACATCTCACTATCT
*SAMT*	ACCATTTTCACATCATTGCCAGAC	CTTCCTCTTTTACCATCAAGTGCTG
*DMAPP*	ATCGGAGAAGAAAGTGAGCGAGAGT	GCCGTGTATCGAAGCAGCAGT
*IPP*	CAGACCATCTCTTTCTCCCACTTTC	GGTCGGAGAGAAGGTGGTAGAGGTA
*GPPS1*	GTTAGCCAACTTTCCATACCATTTC	GAGTGACAACATCATCAAAGAAGGG
*GPPS2*	ATGAAGATGATTAGATTTCGAGTCCAAG	ATAACCAATTTACAACCCCTGACCC
*GPP*	TCTACAGAAAATGGGAGAAAACGAT	TATCTGTTTCTGTCACCAAATCCAC
*LIS*	GGCCAAAGTTAATGAAGTGAGATCC	CGTATATGCCATCGTTGCTGCC
*MYR1*	TTTCACAAAAATTGCCTTCAACCTT	CAAGGTGATGGAGAACTAAAACAAAAC
*FPPS*	TCTTTGTCCAGTTCTTCCAGCGTT	ATCAGTGAAATCAAAGGCGGAATCT
*TER3*	AGAGTTGAATTGCACAGGGTGATAG	GCAGTGGATGTTGTTGATCAGCTC
*TER5*	CTCTCCCTCAGTCTCTTCTCCCTTT	ATCTCCATGCAACATTGGCTACAG

## Supplementary Materials

Supplementary materials can be found at http://www.mdpi.com/1422-0067/19/10/3278/s1.

## Abbreviations

SEM	scanning electron microscope
qRT-PCR	quantitative Reverse Transcript-Polymerase Chain Reaction
*CRC*	*CRABS CLAW*
*FIL*	*FILAMENTOUS FLOWER*
*INO*	*INNER NO OUTER*
*YAB*	*YABBY*
SAMT	*S*-adenosyl-*L*-methionine: salicylic acid carboxyl methyltransferase
DMAPP	dimethylallyl pyrophosphate
IPP	isopentenyl pyrophosphate
GPPS	geranyl diphosphate synthase
GPP	geranyl pyrophosphate
LIS	*S*-linalool synthase
MYR1	myrcenesynthase
FPPS	farnesyl pyrophosphonate synthase
TER	α-terpineol synthase
