# Supplementary material for: Floral Scent Emission from Nectaries in the Adaxial Side of the Innermost and Middle Petals in Chimonanthus praecox
Source: Int J Mol Sci. 2018 Oct 22;19(10):3278. doi: 10.3390/ijms19103278 (PMC6214010; doi:10.3390/ijms19103278)
Supplement: Supplementary file 1 [file ijms-19-03278-s001.pdf]

```
                   *        20         *        40         *        60         *        80         *       100         *       120
CpCRC    : -----------MEFLPS-------SDHVYYVHCSFCNTVLAVGVPRKRMLETVTVKCGHCSNLSFLS--TRSVPQT---HCPDLQLGFQSQGLYSDYMKGQSSSSSSSTSSQLTI---------- :  93
AtCRC    : MNLEEKPTMTASRASPQ-------AEHLYYVRCSICNTILAVGIPLKRMLDTVTVKCGHCGNLSFLT--TTPPLQG---HVS-LTLQMQSFGG-SDYKKGSSSSSSSTSSDQPPS---------- : 102
CpFIL    : ---------MSS--SSAFSVDHLPSSDQLCYVHCNFCDTVLAVSVPCSSLFKTVTVRCGHCTNLLSVN--MRALLLPTPNQLNFS---HSFFSPPHTLLEQIPNPAP--SLLLDQSN------PNQS : 103
AtYABBY1 : ---MSMSSMSSPSSAVCSPDHFSPSDHLCYVQCNFCQTILAVNVPYTSLFKTVTVRCGCCTNLLSVN--MRSYVLPASNQLQLQLGPHSYFNP-QDILEELRDAPSNMNMMMMNQH------PTMN : 114
AtYABBY3 : ---MSSMSMSSSSAPAFPPDHFSSTDQLCYVHCSFCDTVLAVSVPPSSLFKTVTVRCGHCSNLLSVTVSMRALLLPSVSNLGHSFLPPPPPPPPPNLLEEMRSGGQNINMNMMMSHHASAHHPNEH : 123
CpYAB5-1 : ---------MSACVDAAS--------EQLCYIPCNFCNIILAVSVPRSSLFDIVTVRCGHCTNLWSVN--MAAALQS----LSWQEMQTPTFISPDYRLDLGSSSKCN-RVAMLTSV---------- :  93
CpYAB5-2 : ---------MSSCIGATS--------DQVCYIPCNFCNIILAVCVPRSSLFDIVTVRCEHCMNLWSVN--MAAVLQS----LSWQEIQTPTFTSPDYRMNIASSSKCN-KVAMQAS----------- :  92
AtYABBY5 : ---------MANSVMAT---------EQLCYIPCNFCNIILAVNVPCSSLFDIVTVRCGHCTNLWSVN--MAAALQS----LSRPNFQATNYAVPEY----GSSSRSHTKIPSRIST---------- :  89
CpYAB2   : ---------MS--IDVAS--------ERMCYVHCNLCNTILAVSVPFSNLFNIVPIRCGHCANLLSVN--MGSLLQA----FPLQGFQHQNYHCQDKGSDFGSSSKSN-KILMLDST---------- :  91
AtYABBY2 : ---------MS--VDFSS--------ERVCYVHCSFCTTILAVSVPYASLFTLVTVRCGHCTNLLSLN--IGVSLHQTSAPPIHQDLQPHRQHTTSLVTRKDCASSSRSTNNLSENI---------- :  96
AtINO    : --MTKLPNMTTTLNHLFDLP-----GQICHVQCGFCTTILLVSVPFTSLSMVVTVRCGHCTSLLSVN-LMKASFIPLHLLASLSHLDETGKEEVAATDGVEEEAWKVNQEKENSPTTLVSS----- : 113

              *       140         *       160         *       180         *       200         *       220         *       240         *
CpCRC    : -----------------PDVPYVVKPPEKKHRLPSAYNRFMKEEIQRIKTINPKIPHREAFSRAAKNWARSDPHAPNETTLE-----MSKELS-VSFAHENE------------------------ : 172
AtCRC    : -----------------PSPPFVVKPPEKKQRLPSAYNRFMRDEIQRIKSANPEIPHREAFSAAAKNWAKYIPNSPTSITSG-----GHNMIHGLGFGEKK-------------------------- : 181
CpFIL    : LVSTVR---GID---EPPKAPSINRPPEKRQRVPSAYNRFIKDEIQRIKAGNPDITHREAFSAAAKNWAHF-PHIHFGLLPD----QAVKKTSVRQQEG-DEVLIKDGFFS--TANMGITPY---- : 211
AtYABBY1 : DIPSFM---DLHQQHEIPKAPPVNRPPEKRQRVPSAYNRFIKEEIQRIKAGNPDISHREAFSAAAKNWAHF-PHIHFGLVPDN---QPVKKTNMPQQEGEDNMVMKEGFYAPAAANVGVTPY---- : 229
AtYABBY3 : LVMATRNGRSVDHLQEMPRPPPANRPPEKRQRVPSAYNRFIKEEIQRIKAGNPDISHREAFSAAAKNWAHF-PHIHFGLMADH---PPTKKANVRQQEGEDGMMGREGFYG-SAANVGVAHN---- : 240
CpYAB5-1 : ---------------ASNEERTVVNRPPEKRQRIPSAYNQFIKEEIQRIKANNPEISHREAFSTAAKNWAHF-PHIHFGLMLE-----ANNQPKLDEGPEKHLITNAAIYNN--------------- : 184
CpYAB5-2 : ---------------SMNERMMGVNRPPEKRQRVPSAYNQFIKEEIQRIKANNPEISHREAFSTAAKNWAHF-PHIHFGLMLE-----TNNQAKLDEGPEKCLMTKAAVFNK--------------- : 183
AtYABBY5 : ---------------RTITEQRIVNRPPEKRQRVPSAYNQFIKEEIQRIKANNPDISHREAFSTAAKNWAHF-PHIHFGLMLE-----SNKQAKIA-------------------------------- : 164
CpYAB2   : ---------------ENAQPRMLPIPPPEKRPRVPSAYNRFIKEEIQRIKASNPDISHREAFSTAAKNWAHF-PHIHFGLSIE-----GSNQVRIGESLAGEGAQKTPGFY---------------- : 181
AtYABBY2 : ---------------DREAPRMPPIRPPEKRQRVPSAYNRFIKEEIQRIKACNPEISHREAFSTAAKNWAHF-PHIHFGLKLD-----GNKKG--KQLDQSVAGQKSNGYY---------------- : 184
AtINO    : --------SDNEDEDVSRVYQVVNKPPEKRQRAPSAYNCFIKEEIRRLKAQNPSMAHKEAFSLAAKNWAHFPPAHNKRAASDQCFCEEDNNAILPCNVFEDHEESNNGFRERKAQRHSIWGKSPFE : 231
```

Figure S1. Sequence alignment of YABBY proteins in *C. praecox* and *A. thaliana*.

	AfCRC	NnCRC-1	NnCRC-2	AtCRC	Am.trCRC	CpCRC	EcCRC	OsCRC	EsCRC	AtINO	Ny.alINO	Am.trYAB2	CpYAB2	NnYAB2	DlYAB2	AtYAB2	AtYAB5	CpYAB5-1	CpYAB5-2	CsYAB5	AtYABBY1	AtYABBY3	CpFIL	MgFIL	Am.trFIL	NjFIL	Ny.coFIL
AfCRC		68	69	63	56	59	56	50	63	31	38	37	35	36	35	38	37	35	34	48	37	36	36	45	39	37	42
NnCRC-1			100	62	60	67	55	52	62	32	39	40	41	39	38	41	43	40	37	44	37	36	37	44	39	39	42
NnCRC-2				62	60	67	56	52	64	32	40	40	42	40	39	42	44	40	38	44	38	37	38	44	40	40	42
AtCRC					53	59	53	50	57	30	39	40	38	38	42	40	40	40	38	52	36	38	36	53	39	36	51
Am.trCRC						61	54	49	57	31	38	40	37	38	38	37	41	38	36	44	38	39	38	45	40	37	41
CpCRC							57	51	58	33	42	39	38	40	39	42	37	37	37	47	40	41	39	50	40	40	48
EcCRC								56	59	29	38	37	36	37	32	34	41	34	32	43	36	36	35	47	39	38	43
OsCRC									58	31	35	37	36	39	34	38	41	38	35	42	36	36	35	39	37	37	37
EsCRC										33	42	40	38	38	38	39	41	39	37	50	39	36	38	50	43	44	50
AtINO											48	39	37	41	39	42	43	38	38	46	38	36	38	44	38	36	46
Ny.alINO												45	46	48	44	46	52	48	44	49	40	42	43	44	44	43	46
Am.trYAB2													62	61	59	56	58	56	55	65	47	43	49	60	50	50	58
CpYAB2														72	64	57	60	55	55	64	49	47	47	65	51	50	62
NnYAB2															72	64	59	59	59	64	50	49	50	67	52	52	66
DlYAB2																62	58	56	57	67	50	49	49	70	49	48	70
AtYAB2																	53	53	51	67	50	50	52	68	54	51	70
AtYAB5																		72	69	89	54	55	53	77	56	56	76
CpYAB5-1																			81	89	48	47	48	60	51	50	60
CpYAB5-2																				93	49	49	48	63	49	50	62
CsYAB5																					57	59	54	57	62	58	60
AtYABBY1																						62	60	80	58	52	76
AtYABBY3																							59	76	55	52	76
CpFIL																								89	65	61	82
MgFIL																									74	80	81
Am.trFIL																										60	79
NjFIL																											86
Ny.coFIL																											

Table S1 Protein identity analysis by clustalx

Note: Accession numbers details see in section "Sequence alignment and phylogenetic analysis".
